# SpoVG Modulates Cell Aggregation in Staphylococcus aureus by Regulating *sasC* Expression and Extracellular DNA Release

**DOI:** 10.1128/AEM.00591-20

**Published:** 2020-07-20

**Authors:** Qing Zhu, Banghui Liu, Baolin Sun

**Affiliations:** a Department of Oncology, The First Affiliated Hospital, University of Science and Technology of China, Hefei, Anhui, China; University of Naples Federico II

**Keywords:** *Staphylococcus aureus*, SpoVG, transcriptional regulation, cell aggregation

## Abstract

Biofilm formation is involved in numerous Staphylococcus aureus infections such as endocarditis, septic arthritis, osteomyelitis, and infections of indwelling medical devices. In these diseases, S. aureus forms biofilms as cell aggregates interspersed in host matrix material. Here, we have observed that the level of cell aggregation was significantly higher in the isogenic *spoVG*-deletion strain than in the wild-type strain. Reverse transcription-quantitative PCR data indicated that SpoVG could repress the expression of *sasC*, which codes for S. aureus surface protein C and is involved in cell aggregation and biofilm accumulation. Electromagnetic mobility shift assay demonstrated that SpoVG could specifically bind to the promoter region of *sasC*, indicating that SpoVG is a negative regulator and directly represses the expression of *sasC*. In addition, deletion of the SasC aggregation domain in the *spoVG*-deletion strain indicated that high-level expression of *sasC* could be the underlying cause of significantly increased cell aggregation formation. Our previous study showed that SpoVG is involved in oxacillin resistance of methicillin-resistant S. aureus by regulating the expression of genes involved in cell wall synthesis and degradation. In this study, we also found that SpoVG was able to negatively modulate the S. aureus drug tolerance under conditions of a high concentration of oxacillin treatment. These findings can broaden our understanding of the regulation of biofilm formation and drug tolerance in S. aureus.

**IMPORTANCE** This study revealed that SpoVG can modulate cell aggregation by repressing *sasC* expression and extracellular DNA (eDNA) release. Furthermore, we have demonstrated the potential linkage between cell aggregation and antibiotic resistance. Our findings provide novel insights into the regulatory mechanisms of SpoVG involved in cell aggregation and in biofilm development and formation in Staphylococcus aureus.

## Abstract (precis)

This study revealed that SpoVG can modulate cell aggregation by repressing *sasC* expression and extracellular DNA (eDNA) release. Furthermore, we have demonstrated the potential linkage between cell aggregation and antibiotic resistance. Our findings provide novel insights into the regulatory mechanisms of SpoVG involved in cell aggregation and in biofilm development and formation in Staphylococcus aureus.

## INTRODUCTION

Staphylococcus aureus is a major human pathogen and is responsible for a variety of chronic and relapsing infections such as sepsis, osteomyelitis, endocarditis, toxic shock, and infections of implanted devices (1, 2). Bacterial biofilms are the matrix-enclosed structures that comprise bacterial cells, extracellular matrix proteins, carbohydrates, and extracellular DNA (eDNA) and adhere to biological or nonbiological surfaces (3, 4). Biofilm formation is often regarded as a virulence factor and plays a significant role in the chronic infectious process, since bacterial cells in the biofilm can escape host immune attack and resist antibiotic treatment. Biofilm development and formation generally consist of five stages, including attachment, multiplication, exodus, maturation, and dispersal (4). During the attachment stage, planktonic cells adhere to surfaces of biological or nonbiological materials and proliferate into sticky aggregations. However, the successional biofilm growth pattern implies a high variability in mushroom structure development and surface coverage (3). A previous study had pointed to the biological advantage of cell aggregations over single cells during biofilm formation (5). S. aureus cell aggregation is a biological process through which cells bind to matrix proteins and form stable clumps to evade host defenses and to adapt to antibiotic stress. In aggregate communities, S. aureus cells adjust the distribution of their adhesins and surface proteins to promote their tolerance of hazardous environments (5, 6). Biofilm development and formation can be modulated by various regulatory factors such as sigma B (7), the Agr system (7), SaeRS (8, 9), SarA (8), and MgrA (10, 11), but the regulatory mechanisms of cell aggregation remain largely unknown.

In S. aureus, SpoVG is a global transcriptional regulator and binds to the DNA region that contains a characteristic TAATTT/A motif (12). SpoVG can modulate the production of capsule, extracellular nuclease, protease, and lipase (13–15) and the emergence of methicillin and glycopeptide resistance of methicillin-resistant S. aureus (MRSA) and vancomycin-intermediate S. aureus (VISA) (13, 16).

In this study, we found that cell aggregation levels were significantly increased in the S. aureus *spoVG*-deletion strain compared to the wild-type (WT) strain. In addition, reverse transcription-quantitative PCR (RT-qPCR) data identified a potential target gene, *sasC*. By introducing the *spoVG sasC* double mutant, we demonstrated that SpoVG could modulate cell aggregation by repressing *sasC* expression and eDNA release. Our results have further demonstrated that cell aggregation is linked with oxacillin tolerance.

## RESULTS

### The *spoVG*-deletion strain exhibits stronger cell aggregation.

**FIG 1:**
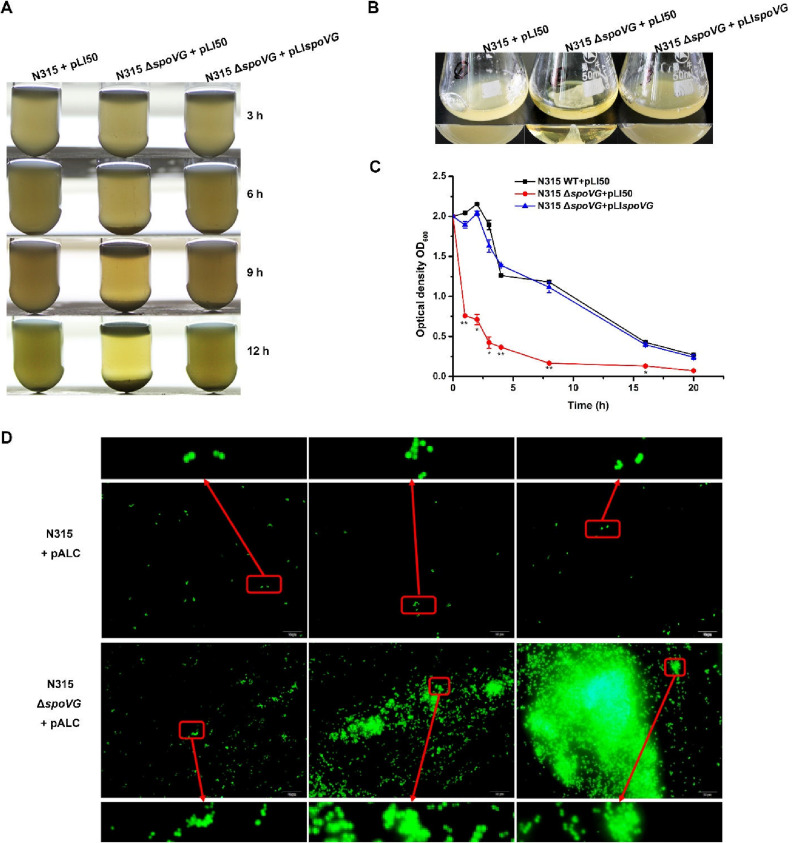
The *spoVG*-deletion strain displayed increased cell aggregation. (A) The WT, *spoVG*-deletion, and *spoVG*-complemented strains were grown in TSB medium. After 3 h, 6 h, 9 h, and 12 h of growth, the *spoVG*-deletion strain exhibited rapid cell clumping, resulting in a clearing of the supernatant. (B) The WT, *spoVG*-deletion, and *spoVG*-complemented strains were grown in flat-bottomed conical flasks. The *spoVG*-deletion strain exhibited a cell net structure after 12 h of incubation at room temperature. (C) After overnight growth, the WT, *spoVG*-deletion, and *spoVG*-complemented strains were adjusted to an OD_600_ of 2 and incubated at room temperature. The OD_600_ of the supernatant was detected within 20 h. Values represent results from three biological replicates ± SEM. Statistical values were determined by the use of the Student's *t* test and the F test to compare variances. *, *P* < 0.05; **, *P* < 0.01. (D) The cell clumps formed in the *spoVG*-deletion strain were visualized using fluorescence microscopy. The fluorescent shuttle plasmid pALC was transformed into the WT and the *spoVG*-deletion strains. The cell aggregations were photographed after growth overnight. (Above) WT strain. (Below) *spoVG*-deletion strain.

During the growth of S. aureus, we found a significant difference in bacterial behavior between the WT and *spoVG*-deletion strains. The *spoVG*-deletion strain exhibited cell clumps after being grown for 3, 6, 9, and 12 h in transparent glass tubes compared with the WT strain, and the alteration could be reversed by *spoVG* complementation (Fig. 1A). When grown in flat-bottomed conical flasks, cells of the *spoVG*-deletion strain gathered together and formed a hard-to-disperse net structure (Fig. 1B). Since the sedimentation of the clumps formed in the *spoVG*-deletion strain resulted in a clearing of the supernatant, a time course of the supernatant of the WT and *spoVG*-deletion strains over 20 h was used to quantify cell aggregation. The *spoVG*-deletion strain displayed a supernatant variation significantly different from the results seen with the WT and *spoVG*-complemented strains (Fig. 1C). In addition, fluorescence microscopy was employed to determine the morphological features of the *spoVG*-deletion strain with the fluorescent shuttle plasmid pALC. As a result, fairly apparent cell clusters were formed in the *spoVG*-deletion strain after growth overnight (Fig. 1D). These data indicate that SpoVG plays a significant role in the regulation of cell aggregation.

### Cell aggregation of the *spoVG*-deletion strain is protease sensitive.

**FIG 2:**
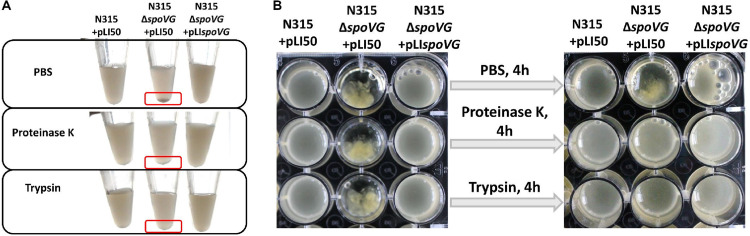
The cell aggregation of the *spoVG*-deletion strain is protease sensitive. The cell aggregation of the *spoVG*-deletion strain was digested with proteinase K and trypsin; PBS was used as a control. (A) Results in small centrifugal tubes. (B) Results in 96-well plates.

To analyze the components of cell aggregation that formed in the *spoVG*-deletion strain, we added proteinase K and trypsin into the cell aggregation culture, and phosphate-buffered saline (PBS) was added as a control treatment. Cell clusters were dissolved after digestion with proteinase K and trypsin (Fig. 2), suggesting that the cell clusters formed in the *spoVG*-deletion strain may involve variations in expression of bacterial surface proteins.

### SpoVG represses *sasC* expression.

**FIG 3:**
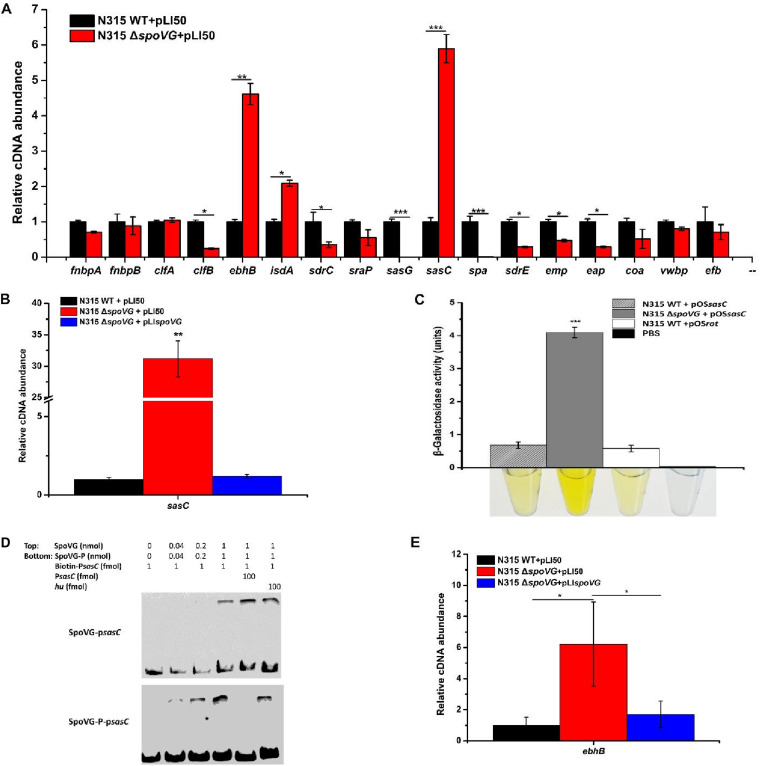
SpoVG represses the transcription of *sasC*. (A) The transcriptional levels of 17 genes encoding extracellular proteins in the WT and *spoVG*-deletion strains were determined by RT-qPCR (OD_600_ of 2). (B) The transcriptional levels of *sasC* in the WT, *spoVG*-deletion, and *spoVG*-complemented strains were determined by RT-qPCR (OD_600_ of 6). (C) The β-galactosidase activity of *sasC* in the WT and *spoVG*-deletion strains. Bacterial cells were collected at an OD_600_ of 6, and the β-galactosidase activity was detected with the substrate ONPG. The *spoVG*-deletion strain carrying pOS*rot* was used as a positive control and PBS as a negative control. (D) EMSA of nonphosphorylated SpoVG (SpoVG) or the hyperphosphorylated SpoVG (SpoVG-P) with the biotin-labeled promoter p*sasC*. The promoter region of *sasC* was amplified by PCR and incubated with purified SpoVG (top) or SpoVG-P (bottom). The unlabeled probe was used as the specific competitor and the unlabeled partial fragment of the *hu* ORF region as the nonspecific competitor. (E) The transcriptional levels of *ebhB* in the WT, *spoVG*-deletion, and *spoVG*-complemented strains were determined by RT-qPCR (OD_600_ of 6). Values represent results from three biological replicates ± SEM. Statistical values were determined by the use of the Student's *t* test and the F test to compare variances. *, *P* < 0.05; **, *P* < 0.01; ***, *P* < 0.001.

SpoVG is a global transcriptional factor and site-specific DNA-binding protein in S. aureus (14–16). To get a deeper understanding of the regulatory role of SpoVG in cell aggregation, we performed RT-qPCR to measure the expression levels of 17 potential target genes at the aggregation-formation stage. These genes included those encoding several members of the microbial surface components recognizing adhesive matrix molecules (MSCRAMMs) and the coagulating proteins in S. aureus, Coa and vWbp. The results showed that the transcriptional levels of 12 genes were altered in the *spoVG*-deletion strain, including 3 upregulated genes (*ebhB*, *isdA*, and *sasC*) and 9 downregulated genes (*clfB*, *sdrC*, *sraP*, *sasG*, *spa*, *sdrE*, *emp*, *eap*, and *coa*). Among these genes, the mRNA levels of *ebhB* and *sasC* were significantly increased in the *spoVG*-deletion strain compared with those in the WT strain (Fig. 3A). The *ebh* gene encodes the giant staphylococcal surface protein (GSSP), which is membrane anchored and protrudes from the cell surface in a fiber-like manner and therefore inhibits cell-cell interactions (17, 18). The *isdA* gene, which encodes IsdA, has been reported to inhibit bacterial biofilm formation. The *sasC* gene encodes S. aureus surface protein SasC, which is involved in cell aggregation and biofilm-accumulation processes (19).

RT-qPCR data indicated that SpoVG may modulate cell aggregation by repressing expression of cell wall proteins, especially that of SasC. Meanwhile, RT-qPCR data showed that the significantly increased level of expression of *sasC* in the *spoVG*-deletion strain could be reversed by *spoVG* complementation (Fig. 3B). To verify the regulatory role of SpoVG in the expression of *sasC*, we constructed a *sasC* promoter-*lacZ* fusion reporter plasmid and determined the levels of β-galactosidase activities in the WT and *spoVG*-deletion strains. As predicted, the promoter activity of *sasC* was increased in the *spoVG*-deletion strain compared with that in the WT strain (Fig. 3C), suggesting that SpoVG is a repressor of *sasC*.

Electrophoretic mobility shift assay (EMSA) was performed to determine whether SpoVG can specifically bind to the *sasC* promoter region. A shifted band was visible after incubation of SpoVG with the biotin-labeled DNA probe containing the *sasC* promoter region (Fig. 3D). This shifted band disappeared when unlabeled *sasC* promoter DNA was added but did not disappear in the presence of unlabeled *hu* DNA as the unspecific competitor, demonstrating that SpoVG can bind to the *sasC* promoter region specifically. Taken together, these results indicated that SpoVG could repress the transcription of *sasC* by directly binding to its promoter region. Furthermore, RT-qPCR data showed that the significantly increased expression of *ebhB* in the *spoVG*-deletion strain could be reversed by the *spoVG* complementation (Fig. 3E).

### The SasC aggregation domain was able to form a superpolymer *in vitro*.

**FIG 4:**
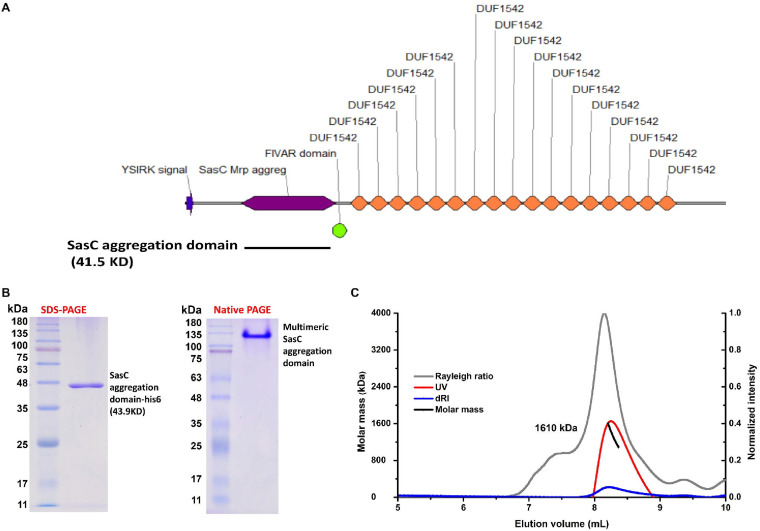
The SasC aggregation domain can form a superpolymer *in vitro*. (A) Domain organization of SasC. SasC consists of the N-terminal signal peptide (represented by a blue arrow), the aggregation domain (represented by a purple diamond), 17 DUF1542 domains (represented by orange diamonds), and the C-terminal LPXTG cell wall-anchored motif. The black line represents the SasC aggregation domain (41.5 kDa). (B) Results of SDS-PAGE (left) and native PAGE (right) analysis of the SasC aggregation domain. (C) Results of FPLC-MALS analysis of the SasC aggregation domain. The molecular weight of the superpolymer SasC aggregation domain is marked.

The SasC protein consists of the N-terminal signal peptide, the aggregation domain, 17 DUF1542 domains, and the C-terminal LPXTG cell wall-anchored motif (Fig. 4A). To investigate the character of the SasC aggregation domain, we expressed this region with a His tag. The SDS-PAGE and native-PAGE results showed that the SasC aggregation domain may exist in polymer form (Fig. 4B). Fast protein liquid chromatography (FPLC) coupled with multiangle light scattering (FPLC-MALS) analysis results revealed that the SasC aggregation domain indeed formed a superpolymer *in vitro*, and the molecular weight was about 1,610 kDa (Fig. 4C), implying that the SasC aggregation domain formed more than 35 polymers.

### Increased *sasC* transcription and eDNA release resulted in cell aggregation in the *spoVG*-deletion strain.

**FIG 5:**
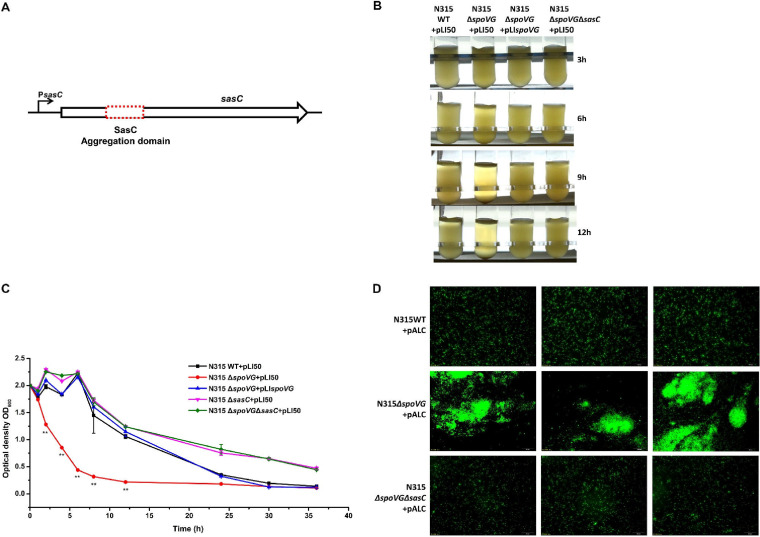
Increased *sasC* transcription in the *spoVG*-deletion strain led to cell aggregation. (A) Structure of *sasC*. The deletion region for *sasC* (SasC aggregation domain) is represented by the red dotted box. (B) After overnight growth, the WT, *spoVG*-deletion, *spoVG*-complemented, and *spoVG sasC* double mutant strains were adjusted to an OD_600_ of 2 and incubated at room temperature for 3, 6, 9, or 12 h. (C) After overnight growth, the WT, *spoVG*-deletion, *spoVG*-complemented, *sasC* mutant, and *spoVG sasC* double mutant strains were adjusted to an OD_600_ of 2 and incubated at room temperature. The OD_600_ of the supernatant was detected within 36 h. Values represent results from three biological replicates ± SEM. Statistical values were determined by the use of Student’s *t* test and the F test to compare variances. **, *P* < 0.01. (D) The cell clumps were visualized using fluorescence microscopy. The fluorescent shuttle plasmid pALC was transformed into the WT, *spoVG*-deletion, and *spoVG sasC* double mutant strains. Cell aggregations were photographed after growth overnight. (Above) WT strain. (Middle) *spoVG*-deletion strain. (Below) *spoVG sasC* double mutant strain.

To investigate the function of SasC in cell aggregation in the *spoVG*-deletion strain, we constructed a SasC aggregation domain deletion in the *spoVG*-deletion strain (Fig. 5A). Compared with the strong evidence of cell clumps seen in the *spoVG*-deletion strain after growth for 3, 6, 9, or 12 h in transparent glass tubes, the SasC aggregation domain deletion in the *spoVG*-deletion strain showed an obvious reduction in the level of cell clumps (Fig. 5B). The optical density at 600 nm (OD_600_) of supernatant of the WT, *spoVG*-deletion, *spoVG*-complemented, and *spoVG sasC* double mutant strains showed that the cell aggregation level was decreased to a large extent in the *spoVG sasC* double mutant strain compared with the *spoVG*-deletion strain (Fig. 5C). Fluorescence microscopy showed similar results (Fig. 5D). These data suggest that SpoVG may modulate cell aggregation by repressing the expression of *sasC*.

**FIG 6:**
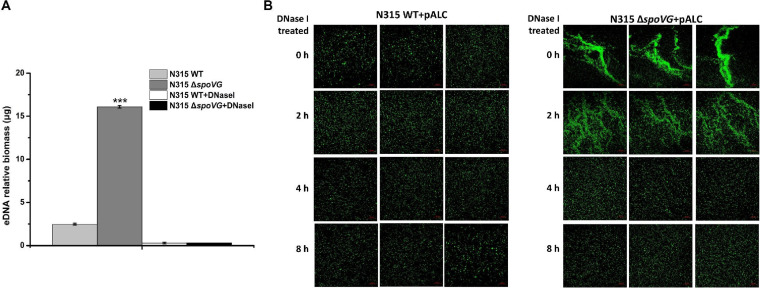
eDNA was involved in the process of bacterial cell aggregation. (A) The level of eDNA release of the WT strain and *spoVG*-deletion strain. DNase I treatment was used as a negative control. (B) The cell clumps processed with DNase I treatment for 0, 2, 4, and 8 h were visualized by fluorescence microscopy. The fluorescent shuttle plasmid pALC was transformed into the WT, *spoVG*-deletion, and *spoVG sasC* double mutant strains. (Left) WT strain. (Right) *spoVG*-deletion strain.

It has been known that eDNA can act as an adhesive and thus strengthen biofilms, which is important for S. aureus biofilm formation. To investigate whether increased cell aggregation of the *spoVG*-deletion strain is eDNA dependent, we determined the amount of eDNA present in the cell aggregation structure. The average amount of eDNA present in cell aggregation of *spoVG*-deletion strain was ∼10-fold that represented in the WT strain, and the amount of eDNA in the *spoVG*-complemented strain was restored to the original level (Fig. 6A), implying a critical role of eDNA in the development of cell aggregation in the *spoVG*-deletion strain. Moreover, after DNase I treatment, the aggregation of the *spoVG*-deletion strain weakened or disappeared (Fig. 6B). These results allow us to conclude that high levels of *sasC* expression and eDNA release can lead to cell aggregation in the *spoVG*-deletion strain.

### Cell aggregation is associated with oxacillin susceptibility and cell survival.

**FIG 7:**
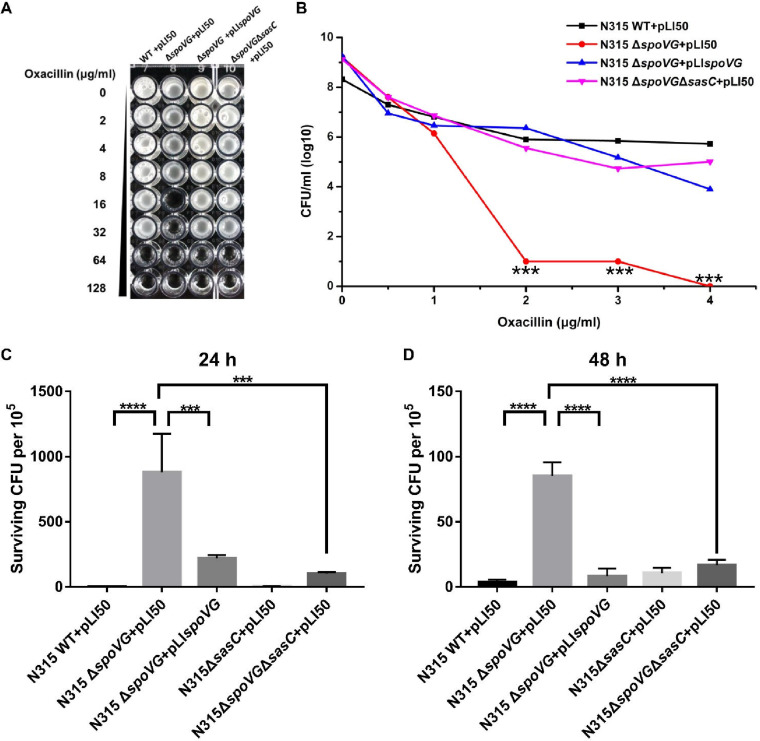
Cell aggregation is associated with oxacillin susceptibility and cell survival. The oxacillin susceptibility of the WT, *spoVG*-deletion, *spoVG*-complemented, and *spoVG sasC* double mutant strains was determined. (A) Results determined by the oxacillin gradient dilution method. (B) Results determined by the plate count method. The WT, *spoVG*-deletion, *spoVG*-complemented, and *spoVG sasC* double mutant strains were grown to the stationary phase and treated with 100× MIC of oxacillin (6.4 mg/ml), and survival rates were determined following 24 or 48 h of incubation. (C) Results from 24 h of incubation. (D) Results from 48 h of incubation. Values represent results from three biological replicates ± SEM. Statistical values were determined by the use of one-way ANOVA and the F test to compare variances. ***, *P* < 0.001; ****, *P* < 0.0001.

The *spoVG*-deletion strain exhibited significantly decreased oxacillin resistance compared with the WT strain, and the phenotype could be restored by *spoVG* complementation (Fig. 7A and B); these results are consistent with our previous study (16). Moreover, the *spoVG sasC* double mutant strain exhibited increased oxacillin resistance compared with that of the *spoVG*-deletion strain (Fig. 7A and B).

We also tested cell survival of the WT, *spoVG*-deletion, *spoVG*-complemented, *sasC* mutant, and *spoVG sasC* double mutant strains in Mueller-Hinton (MH) broth exposed to 6.4 mg/ml of oxacillin (representing approximately 100× MIC for the WT strain) for 24 or 48 h. The *spoVG*-deletion strain exhibited significantly increased drug tolerance after treatment with a high concentration of oxacillin for 24 h compared with the WT strain, and the phenotype could be restored by the *spoVG* complementation (Fig. 7C). Moreover, the *spoVG sasC* double mutant strain exhibited decreased drug tolerance compared with that of the *spoVG*-deletion strain (Fig. 7C). Oxacillin treatment for 48 h showed similar results (Fig. 7D). These results indicated that cell aggregation is tightly associated with oxacillin susceptibility and drug tolerance under conditions of treatment with oxacillin at a high concentration.

## DISCUSSION

Bacteria grow and proliferate depending on their surroundings. In response to certain circumstances, S. aureus can subsist either as single and independent cells or organized in aggregates such as biofilms.

Previous studies have shown that in natural environments and during infections, biofilms are seeded by cell aggregations and individual bacterial cells (5), but an explanation of how aggregation formation is controlled remains elusive. In our experiments, we observed that the *spoVG*-deletion *S aureus* strain displayed a high level of cell aggregation, which disappeared after digestion with protease.

Further, we have demonstrated that SpoVG could modulate cell aggregation by repressing *sasC* expression in S. aureus. SasC is one of the S. aureus surface adhesins that has a typical LPXTG cell wall anchor motif and has been reported to be involved in cell aggregation and biofilm accumulation. Our data revealed that the SasC aggregation domain was able to play a significant role in cell aggregation in the *spoVG*-deletion strain. Furthermore, our data indicated that SpoVG is involved in transcriptional regulation of *ebhB*, and the underlying mechanism requires to be further studied.

S. aureus cells of biofilms are held together in clusters by the electrostatic net formed by eDNA. However, the details of the mechanism by which eDNA is released from S. aureus cells remain unknown. Here, we have revealed a negative regulatory effect of SpoVG on eDNA and further revealed that eDNA plays an important role in the cell aggregation process.

**FIG 8:**
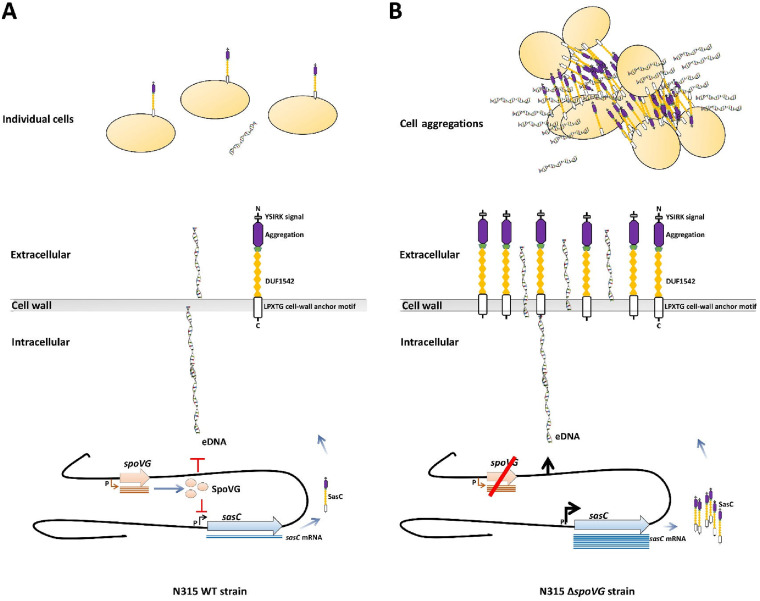
Proposed schema of the SpoVG regulatory mechanism. SpoVG modulates cell aggregation by repressing *sasC* expression and eDNA release. (A) Expression of *sasC* and release of eDNA are repressed by SpoVG in the WT strain. (B) High level of expression of *sasC* and release of eDNA in the *spoVG*-deletion strain, leading to cell aggregation. Black arrows represent the transcription of *sasC*, whereas bars represent repression. Orange arrows represent *spoVG*, and blue circles represent *sasC*. Chains with different domains represent SasC, double-helix structures represent eDNA, and the orange circles represent S. aureus strains.

Overall, this report can facilitate deeper understanding of the regulatory mechanisms of SpoVG involved in cell aggregation in S. aureus, and the function of SpoVG in biofilm formation needs to be further studied. We have also revealed the potential linkage between cell aggregation and antibiotic resistance, and the exact mechanism needs to be further investigated (Fig. 8).

## MATERIALS AND METHODS

### Bacterial strains, plasmids, and growth conditions.

The bacterial strains and plasmids used in this study are listed in Table 1. Escherichia coli Trans1-T1 and BL21(DE3) were grown in Luria broth (LB) medium (Oxoid) with appropriate antibiotics (150 μg/ml ampicillin sodium salt or 50 μg/ml kanamycin sulfate). S. aureus and its derivative strains were grown in tryptic soy broth (TSB) medium (BD) with 15 μg/ml chloramphenicol at 37°C with shaking at 220 rpm (∼16 × *g*). Constructed plasmids were purified from E. coli Trans1-T1 and transformed into S. aureus RN4220 as the initial recipient and then S. aureus strain N315 by electroporation. The media were solidified with 1.5% (wt/vol) agar when needed.

**TABLE 1:** Strains and plasmids used in this study

Strain or plasmid	Relevant genotype^*a*^	Reference or source^*b*^
Strains		
S. aureus RN4220	8325-4, r−	NARSA
S. aureus WT	N315 HA-MRSA, SCC*mec* type II	NARSA
S. aureus Δ*spoVG*	N315 strain deletion of *spoVG*	16
S. aureus Δ*sasC*	N315 strain deletion of the aggregation domain of *sasC*	This study
S. aureus Δ*spoVG* Δ*sasC*	N315 *spoVG sasC* double mutant	This study
E. coli Trans1-T1	Host strain for cloning	TransGen
E. coli BL21(DE3)	Expression strain	TransGen

Plasmids		
pBTs	Shuttle vector, temp sensitive, Amp^r^ Chl^r^	20
pBTsΔ*sasC*	pBTs derivative, for *rot* deletion, Amp^r^ Chl^r^	This study
pETSasC-A	pET28a(+) derivative, with SasC aggregation domain, Kan^r^	This study
pETSpoVG	pET28a(+) derivative, with ORF of *spoVG*, Kan^r^	16
pDuet-Stk1-SpoVG	pRSF-Duet derivative, coexpression of SpoVG and Stk1 kinase domain, Kan^r^	25
pOS1	Shuttle vector, with *lacZ* ORF lacking first 6 amino acids, Amp^r^ Chl^r^	26
pOS*sasC*	POS1 derivative, harboring the *sasC* promoter and 18 bp of *sasC* coding sequence from strain N315, Amp^r^ Chl^r^	This study
pLI50	Shuttle vector, Amp^r^ Chl^r^	27
pLI*spoVG*	pLI50 derivative, harboring ORF of *spoVG* and its promoter, Amp^r^ Chl^r^	16
pALC	Shuttle vector, pALC1484 derivative, harboring ORF of *gfp* and the promoter of the S10 ribosomal gene, Amp^r^ Chl^r^	28

a r−, restriction system negative; HA-MRSA, hospital-acquired MRSA; Kan^r^, kanamycin resistant; Amp^r^, ampicillin resistant; Chl^r^, chloramphenicol resistant.

b NARSA, Network on Antimicrobial Resistance in Staphylococcus aureus.

### Construction of the *sasC* single mutation and *spoVG sasC* double mutation.

To obtain a single mutant with a mutation of the *sasC* gene and a *spoVG sasC* double mutant, the plasmid pBTs and an appropriate protocol was used as described previously (20). Briefly, DNA fragments corresponding to the upstream and downstream regions of *sasC* aggregation domain were amplified by PCR, using S. aureus strain N315 genomic DNA as the template. The PCR products were ligated by overlap PCR to form an up-down fragment, which was purified, digested with KpnI and SalI, and cloned into the temperature-sensitive shuttle plasmid pBTs containing a temperature-sensitive S. aureus origin of replication, a chloramphenicol resistance cassette, and a suicide gene for plasmid maintenance or selection. The resulting plasmid containing the upstream and downstream fragments in tandem was then amplified in E. coli Trans1-T1. The recombinant pBTs was then extracted from E. coli and transformed into S. aureus RN4220 by electroporation at 2.5 kV for modification and subsequently introduced into S. aureus strain N315. The transformants that had allelic replacement of *sasC* were selected on tryptic soy agar (TSA) containing 100 ng/μl anhydrotetracycline (ATC) and were further verified by PCR and DNA sequencing. The *spoVG sasC* double mutant was constructed using a similar strategy by introducing the *sasC* mutant plasmid into the *spoVG*-deletion strain and further confirmed by PCR and DNA sequencing.

### Fluorescence microscopy.

To further measure cell aggregation of S. aureus, fluorescence microscopy was performed. First, cultures of different strains carrying a pALC fluorescence shuttle plasmid with green fluorescent protein (GFP) were grown in TSB for 12 h at 37°C, and then the green fluorescence of the samples excited by blue light were viewed with a fluorescence microscope.

### RNA isolation, cDNA generation, and reverse transcription-quantitative PCR.

For total RNA extraction, the overnight cultures of S. aureus were diluted 1:100 in TSB with appropriate antibiotics and grown to the early exponential phase (OD_600_ of 0.6), midexponential phase (OD_600_ of 2), and stationary phase (OD_600_ of 6). S. aureus cells were collected by centrifugation and processed with 900 μl RNAiso plus (TaKaRa) in combination with 0.1-mm-diameter silica-zirconia beads in a FastPrep-24 automated system (MP Biomedicals Solon). The residual DNA was removed with RNase-free recombinant DNase I (TaKaRa; 5 U/μl). For reverse transcription, cDNA was synthesized with a PrimeScript first-strand cDNA synthesis kit (TaKaRa) using random primers. Reverse transcription-quantitative PCR (RT-qPCR) was performed with SYBR Premix *Ex Taq* (TaKaRa) using a StepOne real-time PCR system (Applied Biosystems) and LC96 real-time PCR system (Roche). The quantity of cDNA was measured by the threshold cycle (2^−ΔΔ^*^CT^*) method with *hu* as the reference gene (21) and the corresponding control sample as the run calibrator. The primers used in this study are listed in Table 2. All the RT-qPCR assays were repeated at least three times.

**TABLE 2:** Primers used in this study

Primer	Sequence (5′–3′)^*a*^	Application
*sasC*-up-F-kpnI	GCGggtaccGAGAACAGACAACCAGGA	*sasC* cell aggregation domain deletion
*sasC-u*p-R	TGTAATTGCTACTGGTGCAC	*sasC* cell aggregation domain deletion
*sasC-d*own-F	GTGCACCAGTAGCAATTACAAATCAAATGCAACATACGTT	*sasC* cell aggregation domain deletion
*sasC-d*own-R-salI	GCGgtcgacTTGGCGTTATCAACATCA	*sasC* cell aggregation domain deletion
p*sasC-F*-EcoRI	GCGgaattcTCCGGATAATTTAAATTCAT	pOS*rot*
p*sasC*-6aa-R-BamHI	GCGggatccGGTTTCTTTAACAAATTCAT	pOS*rot*
probe-p*sasC*-F	TCCGGATAATTTAAATTCAT	EMSA
probe-p*sasC*-Biotin-R	TTTCTTTAACAAATTCAT	EMSA
RT-*sasC*-f	GCACCAGTAGCAATTACAG	RT-qPCR
RT-*sasC*-r	AGACAGCACTTCGTTAGG	RT-qPCR
*sasC*-aggre-NdeI-F	GGAATTCcatatgTACACGCCGACAACTGATCC	*sasC* cell aggregation domain expression
*sasC*-aggre-XhoI-R	CCGctcgagTTATCTATCAACCTCGGCTTGTA	*sasC* cell aggregation domain expression

a Lowercase letters indicate restriction sites. Underlined letters indicate complementary sequences used for overlap PCR ligation.

### Construction of the LacZ reporter vector.

To construct reporter plasmid pOS*sasC*, the DNA fragment containing the *sasC* promoter region was amplified from S. aureus strain N315 genomic DNA using primers listed in Table 2. The fragment was digested with BamHI and EcoRI and cloned into the shuttle vector pOS1. The reporter plasmid was transformed first into S. aureus RN4220 for modification and then into the WT and *spoVG*-deletion strains.

### β-Galactosidase activity assay.

Analysis of β-galactosidase activity was performed as previously described (22). For β-galactosidase assay with o-nitrophenyl-β-d-galactopyranoside (ONPG) as the substrate, the WT and *spoVG*-deletion strains were grown to the stationary phase, centrifuged, and then resuspended in 100 μl of ABT-LSA buffer (60 mM K_2_HPO_4_, 40 mM KH_2_PO_4_, 100 mM NaCl, 0.1% Triton X-100, 50 μg/ml lysostaphin). The samples were maintained under shaking conditions at 37°C until thoroughly lysed. Then, 100 μl ABT buffer and 50 μl ONPG were added to initiate the reaction. The samples were incubated at 37°C until a yellow color became apparent, and 1 ml Na_2_CO_3_ (1 M) was then added to stop the reaction. Sample absorbance was read at 420 nm, and units were calculated using the following formula: units = (1,000 × OD_420_)/(*T* × *V* × OD_600_) (where *T* [measured in minutes] was the incubation time and *V* [in milliliters] was the volume of culture used in the assay). The assays were repeated at least three times.

### Electrophoretic mobility shift assay.

The biotin-labeled DNA fragment p*sasC* containing *sasC* promoter region was amplified from S. aureus strain N315 genomic DNA using primers listed in Table 2. The amplified biotin-labeled p*sasC* fragment was incubated at 25°C for 30 min with various amounts of SpoVG-P and SpoVG in incubation buffer (50 mM Tris-HCl, 300 mM NaCl, pH 8.0) for electrophoretic mobility shift assay (EMSA). After incubation, the mixtures were electrophoresed in a 4% native polyacrylamide gel in 1× Tris-borate-EDTA (TBE) buffer and then transferred to a nylon membrane in 0.5× TBE buffer. The band shifts were detected using a chemiluminescent nucleic acid detection module kit (Thermo Fisher) and were imaged with an ImageQuant LAS 4000 system (GE Healthcare). The unlabeled fragment of promoter was added as the specific competitor (SC). The unlabeled ∼100-bp DNA fragment derived from the open reading frame (ORF) of *hu* was added as the nonspecific competitor (NC).

### Purification and detection of eDNA.

Purification and detection of eDNA were performed as previously described (23). Samples of an overnight bacterial culture were collected by centrifugation, and the samples were first treated with 5 μg/ml proteinase K at 37°C for 1 h. Following treatment, bacterial samples were centrifuged and the supernatant was filtered using a 0.22-μm-pore-size polyether sulfone membrane to remove the bacterial cells. The extracellular DNA (eDNA) was extracted through the use of a phenol-chloroform-isoamyl alcohol DNA extraction method. The aqueous phase was added with sodium acetate at a final concentration of 0.3 M and 0.6× volume of isopropanol. The eDNA precipitation was washed twice with 75% (vol/vol) ethanol, air-dried, and dissolved in 500 μl nuclease-free water.

### Cloning, expression, and purification of recombinant SasC aggregation domain and FPLC-MALS.

The DNA region encoding the SasC aggregation domain was amplified by PCR using S. aureus strain N315 genomic DNA as the template. The corresponding PCR product was digested by NdeI and XhoI and was then ligated into the pET-28a (+) vector, generating plasmid pETSasC-A. The resulting plasmid was verified by DNA sequencing and then transformed into E. coli BL21(DE3), and the transformant was grown in LB medium with 50 μg/ml kanamycin at 37°C to an OD_600_ of 0.4 to 0.6 and then induced with 0.5 mM isopropyl-β-d-1-thiogalactopyranoside at 37°C for additional 3 h. The cells were then harvested, resuspended in lysis buffer (50 mM Tris-HCl, 300 mM NaCl, pH 8.0), and lysed by sonication. The His-tagged SasC aggregation domain protein was purified by the use of nickel-nitrilotriacetic adid (Ni-NTA) resin (Qiagen) and Superdex 200 Increase 10/300 GL column (GE Healthcare). SDS-PAGE and the bicinchoninic acid (BCA) assay were used to analyze the protein purity and concentration, respectively. The accurate molar mass of protein was measured by FPLC-MALS (Wyatt Technology).

### Oxacillin susceptibility assay.

The oxacillin susceptibility assay was performed as described by Clinical and Laboratory Standards Institute. Bacterial strains were serially diluted and plated on Mueller-Hinton agar with 2% NaCl containing increasing concentrations of oxacillin. CFU counts were determined after overnight incubation at 37°C.

### Drug tolerance assay.

The drug tolerance assay was performed as previously described (24). Bacterial strains were grown to the early exponential and stationary phase, and the cultures were serially diluted and plated on agar to determine the initial CFU. For drug tolerance detection, bacterial strains (early exponential and stationary phase) were treated with oxacillin for 24 and 48 h at 100× MIC (6.4 mg/ml). Following treatment, cultures were collected, washed with 0.9% NaCl to remove the oxacillin, and then serially diluted and spot plated to determine the posttreatment CFU. The drug tolerance was determined as follows: posttreatment CFU/initial CFU.

### Statistical analyses.

All experiments were performed in biological triplicate. Values are from three biological replicates ± SEM (standard errors of the means). Statistical values were determined by the use of Student’s *t* test (for two groups), analysis of variance (one-way analysis of variance [ANOVA], for more than two groups), and the F test to compare variances, with a *P* value of <0.05 considered significant (*, *P* < 0.05; **, *P* < 0.01; ***, *P* < 0.001; ****, *P* < 0.0001).
